# Epitranscriptome Analysis of Oxidative Stressed Retinal Epithelial Cells Depicted a Possible RNA Editing Landscape of Retinal Degeneration

**DOI:** 10.3390/antiox11101967

**Published:** 2022-09-30

**Authors:** Luigi Donato, Concetta Scimone, Simona Alibrandi, Sergio Zaccaria Scalinci, Carmela Rinaldi, Rosalia D’Angelo, Antonina Sidoti

**Affiliations:** 1 Department of Biomedical and Dental Sciences and Morphofunctional Imaging, Division of Medical Biotechnologies and Preventive Medicine, University of Messina, 98125 Messina, Italy; 2 Department of Biomolecular Strategies, Genetics and Cutting-Edge Therapies, I.E.ME.S.T., 90139 Palermo, Italy; 3 Department of Chemical, Biological, Pharmaceutical and Environmental Sciences, University of Messina, 98125 Messina, Italy; 4 DIMEC (Department of Medical and Surgical Sciences), University of Bologna, 40121 Bologna, Italy

**Keywords:** RNA editing, RNA-Seq, RPE, A2E, oxidative stress, IRDs

## Abstract

Oxidative stress represents one of the principal causes of inherited retinal dystrophies, with many related molecular mechanisms still unknown. We investigated the posttranscriptional RNA editing landscape of human retinal pigment epithelium cells (RPE) exposed to the oxidant agent N-retinylidene-N-retinyl ethanolamine (A2E) for 1 h, 2 h, 3 h and 6 h. Using a transcriptomic approach, refined with a specific multialgorithm pipeline, 62,880 already annotated and de novo RNA editing sites within about 3000 genes were identified among all samples. Approximately 19% of these RNA editing sites were found within 3′ UTR, including sites common to all time points that were predicted to change the binding capacity of 359 miRNAs towards 9654 target genes. A2E exposure also determined significant gene expression differences in deaminase family ADAR, APOBEC and ADAT members, involved in canonical and tRNA editing events. On GO and KEGG enrichment analyses, genes that showed different RNA editing levels are mainly involved in pathways strongly linked to a possible neovascularization of retinal tissue, with induced apoptosis mediated by the ECM and surface protein altered signaling. Collectively, this work demonstrated dynamic RNA editome profiles in RPE cells for the first time and shed more light on new mechanisms at the basis of retinal degeneration.

## 1. Introduction

In addition to transcription, posttranscriptional RNA modifications (PTMs) contribute to extending function and diversity of transcripts. One of the most interesting PTMs is RNA editing, which increases biologically relevant diversity of transcripts or protein isoforms [1]. RNA editing is a recently discovered mechanism that modifies the RNA sequence itself without changing its genomic DNA (gDNA) through nucleotide insertion, deletion, and substitution [2]. In contrast to DNA modifications, for which a role in gene expression has already been established, the biological significance of chemical alterations, including isomerization and editing of canonical bases, has not been fully characterized [3]. Currently, it is known that such modifications may determine transcriptome plasticity and diversity along with changing amino acids, affecting RNA stability, modulating the nuclear retention of RNAs, and impacting alternative splicing (AS) [4]. RNA editing by base conversion can induce different biological effects depending on RNA type (mRNA or non-coding RNA), region (5′ or 3′ untranslated region (UTR)), coding sequence (CDS) or intron involved in the modification [5]. Growing evidence suggests that RNA editing events in UTRs can alter gene expression and regulation and, while most editing occurs in Alu repeats [6], many others can also affect protein coding regions, leading to amino acid replacements with several functional effects [7,8].

Presently, two major canonical RNA editing types in mammals are known, including adenosine-to-inosine (A-to-I) editing (the most frequent in vertebrates) under the catalysis of adenosine deaminase acting on RNA (ADAR) proteins [9], and cytidine-to-uridine (C-to-U) RNA editing under the catalysis of cytidine deaminases belonging to the apolipoprotein B editing catalytic polypeptide-like (APOBEC) family [10]. A-to-I RNA editing in mammals modulates innate immune response, and alterations have been implicated in the pathogenesis of many diseases, such as autoimmune and inflammatory tissue injury, as well as diverse tumors [10]. Interestingly, RNA-editing events can regulate brain physiology within the mammalian central nervous system (CNS), particularly functions related to neurotransmission [11]. Accordingly, dysregulation of the RNA editing process has been reported to be associated with several human neurological, neurodegenerative, and psychiatric disorders, such as autism spectrum disorder, amyotrophic lateral sclerosis (ALS) and schizophrenia [12]. 

RNA editing can also occur in the form of U-to-C, G-to-A and others, collectively called “alternative RNA editing”. U-to-C was initially identified in the mRNA of Wilm’s tumor 1 (WT1) human transcript [13], while G-to-A was identified in the heterogeneous nuclear ribonucleoprotein K (hnRNP K) protein of colorectal cancer and surrounding tissues [14]. However, the exact mechanism of these RNA editing types is still under investigation, and there is also disagreement regarding the prevalence of such non-canonical RNA editing [15]. 

Furthermore, primary transcripts of tRNA genes, tRNA precursors, are heavily processed (e.g., removal of intronic sequences, trimming of both 5′ and 3′ termini, modification of a large number of tRNA bases) before they fold into functional mature tRNAs. Thus, it is not surprising that editing events can also occur in tRNA, and such modifications are catalyzed by the ADAT (adenosine deaminase acting on tRNA) family enzyme. For example, tRNA genes with defectively paired or incomplete acceptor stems can be transcribed into tRNA precursors whose acceptor stem is reconstructed in a template dependent editing pathway. Other examples of editing involve the insertion or deletion of single bases or short sequences in the tRNA precursor [16]. 

MicroRNAs (miRNAs) and their binding sites may also be processed by ADARs to control miRNA biogenesis and regulation activity [17].

Such a complex scenario has highlighted the need for novel technologies to investigate the effects of RNA editing events, and the advancement of computational methods has enabled the elucidation of many RNA editing sites in humans [18]. Today, RNA-Seq is the gold standard approach to discover RNA editing candidates in whole eukaryotic genomes [19]. While the identification of editing sites is, in theory, quite simple, it represents a computational challenge as true RNA editing events must be distinguished from genome-encoded single-nucleotide polymorphisms (SNPs) and from technical artefacts produced by sequencing or read-mapping errors [20]. These controversies have highlighted the relevance of accurate RNA-Seq mapping to the human transcriptome [21], which can reduce false-positive RNA editing events and increase the sensitivity for detection of true events. RNA-Seq mapping algorithms were initially evaluated to establish gene expression levels but were not optimized for detecting RNA editing events. For example, when sequencing reads end near splice junctions or true RNA editing events, soft-clipped mappings may be produced, hampering detection of editing events. It is also difficult to ensure accurate mapping to paralogs or expressed pseudogenes [22]. 

In the present work, for the first time we describe the RNA editing event landscape of retinal pigment epithelium (RPE) cells in a physiological condition versus exposition to the oxidant agent N-retinylidene-N-retinyl ethanolamine (A2E). A2E is a toxic bis-retinoid deriving from the condensation and oxidation of 11-*cis*-retinaldehyde (11-*cis*-RAL) or all-*trans*-retinal retinaldehyde (all-*trans* RAL) [23,24], capable of reproducing an oxidative stressed microenvironment typical of many retinal dystrophies [25,26]. Even though A2E itself is characterized by a low phototoxic effect, human RPE cells treated with it and then exposed to blue light resulted in a significant decrease in cell viability in vitro [27,28]. Human RPE (H-RPE) is comprised of hexagonal cells organized in a monolayer that is densely packed with pigment granules, situated between the neuroretina and the choroids, and is of neuroectodermal origin. H-RPE cells play a critical role in visual function and photoreceptor viability and, as highlighted in our previously published works, treatment with A2E may determine significant differences in gene expression and splicing events, involving many biological pathways recently related to retinal degeneration [29,30]. An approach based on the use of multiple alignment algorithms was exploited, trying to reduce mapping artifacts in RNA-Seq and improve the detection of RNA editing. In particular, the combined use of the newest resources in RNA editing event discovery with consolidated experimental basis gave strength and reliability to this work.

## 2. Materials and Methods

### 2.1. Cell Culture and Photo-Oxidation Induction

Human RPE-derived cells (H-RPE—Human Retinal Pigment Epithelial Cells, Clonetics™, Lonza, Basel, Switzerland, cat. n°: 00194987) were cultivated following previously described protocols [31]. Cells are cryopreserved, primary cells that are packaged at passage 2 and contain ≥500,000 cells per vial. In detail, H-RPE cells were grown in T-75 flasks containing RtEGM™ Retinal Pigment Epithelial Cell Growth Medium BulletKit^®^ (Clonetics™, Lonza, Basel, Switzerland, cat. n°: 00195409) with 2% *v*/*v* fetal bovine serum (FBS), 1% penicillin/streptomycin, and then incubated at 37 °C with 5% CO_2_. H-RPE cells were then plated into 96-well plates (4 × 10^4^ cells/well) and cultivated for 24h to reach confluence before A2E (AptaBio, Yongin, Korea) addition, in a final concentration of 20 μM for 6h and following rinsing with medium. A2E was synthesized by incubating ethanolamine (4.75 mg, 77.5 μmol) and all-*trans*-retinal (50 mg, 176 μmol) in ethanol (3 mL) at room temperature for 2 days in the dark. A2E was then purified by silica gel chromatography, and purity was confirmed by thin-layer chromatography (TLC) and high-performance liquid chromatography (HPLC). HPLC-purified A2E was exposed to room light for varying time periods to provide mixtures of A2E-related photoisomers. Control cell groups were incubated without A2E. Confluent cultures were transferred to PBS supplemented with Mg^2+^, Ca^2+^ and glucose, before exposure to blue light emitted by a tungsten-halogen source (470 ± 20 nm; 0.4 mW/mm^2^) for 30 min to induce phototoxicity of A2E and subsequent incubation at 37 °C, as already described by confirmed protocols [32,33,34]. A2E quantity in cell cultures post blue light induction was then confirmed by HPLC. The first 3 generations of subcultured RPE cells were used in this experiment. 

### 2.2. MTT Assay

Cell viability was determined by mitochondrial-dependent reduction of methylthiazolyldiphenyl-tetrazolium bromide (MTT) (Sigma-Aldrich, St. Louis, MO, USA) to formazan-insoluble crystals, following a previously described protocol [31]. Results were expressed as a percentage of viable cells normalized with control conditions without A2E.

### 2.3. Total RNA Sequencing

Whole RNA was isolated by TRIzol^TM^ Reagent (Invitrogen^TM^, ThermoFisher Scientific, Waltham, MA, USA) following manufacturer’s protocol, and quantified at Qubit 2.0 fluorimeter. The RNA-Seq samples consisted of 3 factor groups, represented by H-RPE cells, before treatment with A2E and at 4 following time points of 1 h, 2 h, 3 h and 6 h, chosen on the basis of our precedent experiences [31]. These results highlighted that in wider time intervals, the death rate of oxidative stressed cells might be so high as to invalidate any following data analyses. Libraries were generated using 1 µg of total RNA by the TruSeq Stranded Total RNA Sample Prep Kit with Ribo-Zero H/M/R (Illumina, San Diego, CA, USA), according to manufacturer’s protocols. Sequencing runs were performed on a HiSeq 2500 Sequencer (Illumina, San Diego, CA, USA), using the HiSeq SBS Kit v4 (Illumina, San Diego, CA, USA). The experiment was repeated three times.

Obtained raw sequences were filtered to remove low-quality reads (average per base Phred score <30) and adaptor sequences. The quality of analyzed data was checked using FastQC (v.0.11.9) (Babraham Institute, Cambridge, UK, https://www.bioinformatics.babraham.ac.uk/projects/fastqc/, accessed on 17 August 2022) and QualiMap (v.2.2.1, accessed on 17 August 2022) [35], while trimming was realized by Trimmomatic (v.0.39) (Max Planck Institute of Molecular Plant Physiology, Golm, Germany, accessed on 17 August 2022). 

In the first phase, filtered reads were aligned using a combination of mappers; in detail, CLC Genomics Workbench v.22.0 (https://digitalinsights.qiagen.com/products-overview/analysis-and-visualization/qiagen-clc-genomics-workbench/, accessed on 17 August 2022), BWA_MEM v.2.2.1 (accessed on 17 August 2022) [36], HISAT v.2.2.1(accessed on 17 August 2022) [37], TopHat 2.1.1 (accessed on 17 August 2022) [38], STAR 2.7.10 (accessed on 17 August 2022) [39] and RASER 0.5.2 (accessed on 17 August 2022) [40] against the Homo sapiens genome hg38 and the RNA database v.105, on the Ensembl database. 

After the previous individual mappings, the best record for each read from among the six available was chosen according to the following rules: (1) mapped reads preferred to unmapped; (2) among mapped reads, those characterized by more matches are always better than those with fewer; (3) among mapped reads with the same number of matches, records presenting fewer indels were preferred.

In the presence of multiple candidates, the best-read pair was chosen based on the following criteria: (1) pairs on the same chromosome are preferred to pairs mapping on different chromosomes; (2) pairs that are closer (on a log_10_ scale, with integer granularity) were considered better; (3) pairs in forward-reverse orientation were preferred; (4) pairs from the same aligner and database combination were ultimately preferred.

Finally, the individual aligned BAM files were sorted and merged, duplicates were removed, and the files were indexed using Picard tools SortSam, MergeSamFiles and Mark-Duplicates. Recalibration of the aligned reads was then performed by the Genome Analysis Toolkit (GATK) (v.4.1.3.0) (https://software.broadinstitute.org/gatk/ (accessed on 17 August 2022)).

Details on each aligner settings are available in Table 1. Raw data are available upon request.

### 2.4. RNA Editing Event Detection and High-Confidence Filtering

Once the alignment phase was concluded, the RNA editing detection step was performed. The analytic pipeline started with variant calling using the fixed ploidy variant detection in the CLC Genomics Workbench suite, reporting variants with >95% probability. The algorithm behind the Fixed Ploidy Variant Caller combines a Bayesian model (examining posterior probabilities) with a maximum likelihood approach. Found variants were then annotated by the ANNOVAR v.20211019 (accessed on 17 August 2022) tool and included databases, limiting analysis to single nucleotide variants (SNVs) and removing false editing events due to germline variants, paralogous mapping and homopolymer regions. Furthermore, to rescue low-confidence editing events for which editing was not detected de novo, we compared identified editing sites with ones annotated in the RADAR (accessed on 17 August 2022) [41] and REDIportal (accessed on 17 August 2022) [41] databases, using the REDItools (accessed on 17 August 2022) [42] and SPRINT (accessed on 17 August 2022) [43] tools. De novo editing sites were filtered according to the Bonferroni-adjusted *p*-value and only those showing a *p*-value of < 0.05 were selected for downstream analysis. However, while loci spanned by annotated editing sites are indicated in the REDIportal output file, this information is not provided for the de novo ones. Thus, the Variant Effect Predictor (VEP) tool of the Ensembl Genome Browser (https://www.ensembl.org/Homo_sapiens/Tools/VEP?db=core, accessed on 17 August 2022) was used for annotation of the de novo editing sites as well as for classification, according to functional class [44]. In order to process downstream analysis with the same data format, the already annotated editing sites were also run at the VEP tool. According to VEP prediction, only editing modifications showing “HIGH”, “MODERATE” and “MODIFIER” impact on gene expression or function were considered for downstream analysis. 

Using the BamDeal toolkit (https://github.com/BGI-shenzhen/BamDeal, accessed on 17 August 2022), identified RNA editing events were filtered on the basis of high-confidence, defined by the following criteria: (1) an editing site showing read coverage of >100 required at least 3 mutant reads to be considered edited with high confidence; (2) sites with 20–99 reads of coverage needed at least 2 mutant reads; (3) for sites with <20 reads of coverage, only 1 mutant read was required to consider them as high confidence. Such thresholds were based on analysis of adjacent control sites that were within 2 base pairs upstream or downstream of the identified RNA editing sites in all RNA-Seq sample data, determining the rate of true-positive editing, quantifying the background error mutation rate. The editing level of each site was calculated as Gs/As + Gs. The overall editing level of each sample was calculated as the number of Gs divided by the total number of As + Gs at all editing sites.

### 2.5. Comparison of RNA Editing between Treated Samples and Controls

After selection of edited loci based on impact on the spanning editing sites, these loci were clustered according to the differential distribution of the editing sites between treated and control samples. In detail, three groups of genes were considered: (1) genes edited in both 3 h and 6 h treated samples but showing different editing events and frequency; (2) genes only edited in 3 h treated samples; (3) genes only edited in 6 h treated samples. For each editing site, editing ratio between treated samples and/or control was calculated. Frequencies of editing events were calculated by IBM SPSS Statistics 26.0 software (accessed on 17 August 2022)) [45].

### 2.6. Functional Enrichment of Differential Edited Genes and miRNAs

In order to explore the effect of RNA editing on miRNA targeting, the flanking 13 bp-long sequence of the editing site (6 bp each side) and the seed of known miRNA were compared. A site was regarded as a candidate binding site if any 7 bp sequence could be completely complementary to a known miRNA seed [46]. Subsequently, to identify pathways in which genes and miRNAs with different editing sites (DESs) are involved, enrichment analysis was performed with the Enrichr engine [47], which currently contains a collection of ~400,000 annotated gene sets organized into ~300 gene-set libraries, and the FunRich v.3.1.3 tool (accessed on 17 August 2022) [48]. Among them, Gene Ontology (GO) [49], Kyoto Encyclopedia of Genes and Genome (KEGG) [50], InterPro [51], Reactome [52], Human Protein Atlas [53], UniProt [54], IntAct [55], miRbase [56], Ensembl [57] and HGNC [58] databases provided hierarchical relationships for the gene products distinguishing biological process, molecular function and cellular component. Only results showing a hypergeometric test with Bonferroni-adjusted *p*-value of <0.05 were considered. 

### 2.7. ADAR Expression Level Analysis

From previous mappings, the paired end reads were categorized and assigned to the genes, according to abundances, using the expectation-maximization (EM) algorithm [59]. Shortly after, differential expression analysis between control and treated samples, after 3 h and 6 h, was carried out, applying the Empirical analysis of the DGE (EDGE) statistical algorithm [60] by CLC Genomics Workbench, as already described in other papers [31]. The ADAR family encoding genes uniquely identified in the RPE cells with at least 5 unique gene reads, showing a fold change (FC) > 1 (up-regulated) or between 0 and 1 (down-regulated), with Bonferroni-adjusted *p*-values of <0.05 filtered and evaluated. Furthermore, as the FCs were linear, they were replaced by log2 values to make the variation more noticeable (for instance, 2-fold down-regulation is indicated by a value of −1 instead of 0.5).

### 2.8. Data Validation by qRT-PCR

The different deaminase dysregulated mRNAs previously identified were validated by quantitative Real-Time-Polymerase Chain Reaction (qRT-PCR) to confirm RNA-Seq data results, following an already described protocol [31]. Each reaction foresaw six replicates, considering all analyzed time points (18 samples), and the average threshold cycle (Ct) was calculated for each replicate. mRNA expression was computed in relation to expression level of endogenous control β-actin, and the relative expression of genes was calculated using the 2^−ΔΔCt^ method [61]. Statistical significance was determined by analysis of variance between groups (ANOVA), followed by the Bonferroni post-hoc test. Finally, a linear regression analysis was performed to check correlation of FC of the gene expression ratios between RNA-Seq and qRT-PCR. Adjusted *p*-values of < 0.05 were considered statistically significant. All statistical analyses were performed using IBM SPSS 26.0 software (accessed on 17 August 2022) [45]. The list of primers is available in Table S1.

## 3. Results

### 3.1. MTT Assay Showed Increased RPE Cell Death in A2E Treated Samples

**Figure 1:**
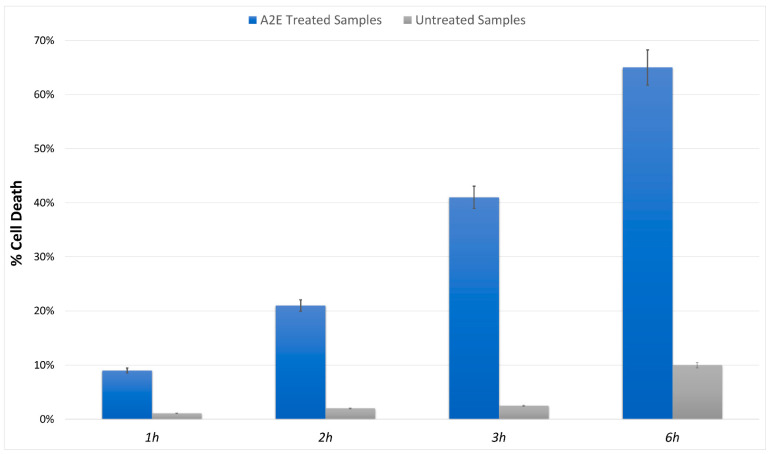
MTT determination of A2E treatment in RPE cells. Cell death was assessed at evaluated time points (1 h, 2 h, 3 h and 6 h) in A2E stressed samples (blue bars) compared to basal untreated group (grey bars). Results are shown as mean ± standard error of mean (n = 3). *p*-value < 0.05.

The MTT cell viability assay highlighted a significant, different trend in RPE-treated cells versus control. The addition of A2E to cultures led to a dose-dependent increase in cell death percentage (Figure 1).

### 3.2. Sequencing Analysis and Mapping Statistics

RNA sequencing carried out on Illumina NovaSeq 6000 (Illumina, San Diego, CA, USA) yielded a total of approximately 96 million quality reads (mean mapping quality = 29) with a percentage of 68% uniquely mapped. About 16,200 genes and 70,000 transcripts were identified out of 60,609 and 227,462 reference counterparts, respectively, considering the whole human transcriptome. The annotated reference assembly v.40 (GRCh38.p13) was downloaded from GeneCode FTP server (ftp://ftp.ebi.ac.uk/pub/databases/gencode/Gencode_human/, accessed on 17 August 2022). All previous mapping statistics were based on average values calculated for all three replicates in each time point. 

### 3.3. Identification of RNA Editing Sites in A2E Treated RPE Cells 

**Figure 2:**
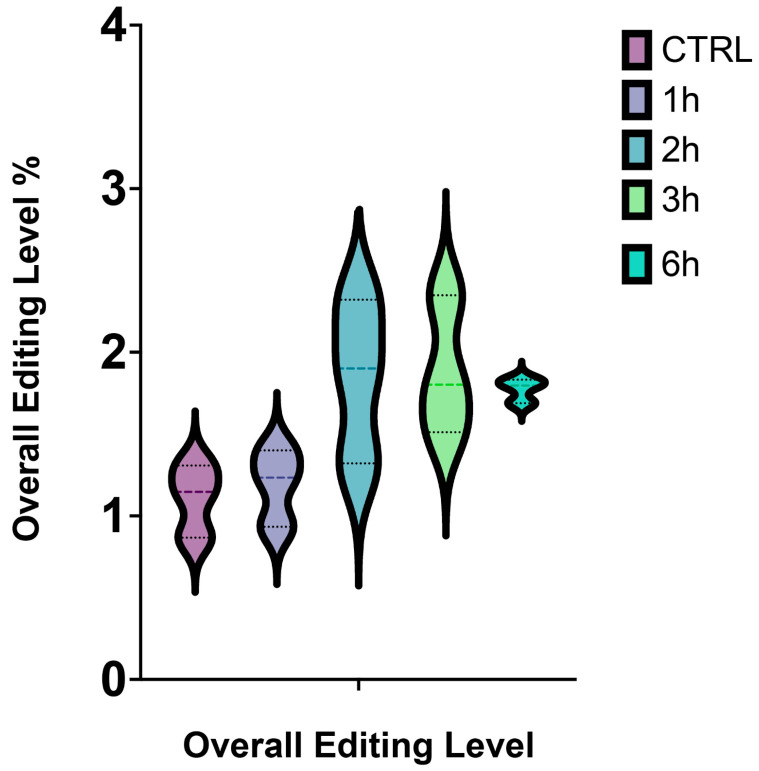
Overall editing level of RPE cells stressed by A2E. The simplified violin plot highlights the different distribution of editing levels throughout the whole treatment.

Altogether, we observed that the overall editing level reached the highest value after 3 h from A2E treatment, while the lowest after 6 h (Figure 2).

**Figure 3:**
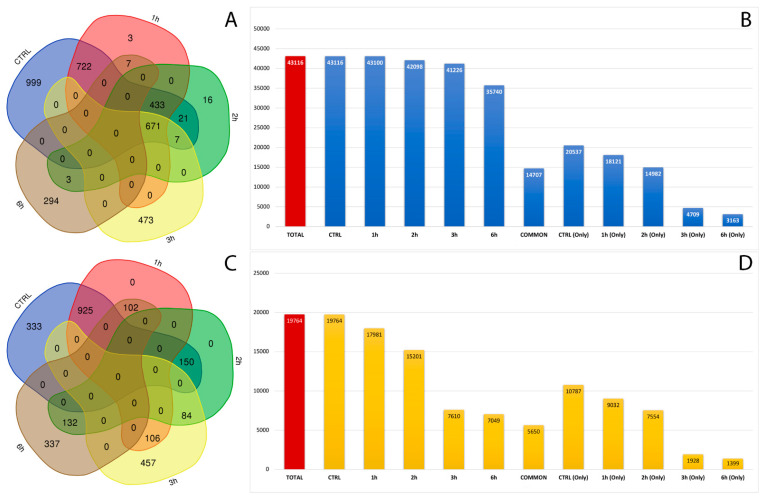
Number of genes and editing sites during treatment stages. (**A**) Number of unique or common genes with already known editing sites in RPE samples at considered time points. (**B**) Number of known editing sites in RPE samples at considered time points. (**C**) Number of unique or common genes with de novo editing sites in RPE samples at considered time points. (**D**) Number of de novo editing sites in RPE samples at considered time points.

After filtering low-quality reads and adapter sequences and by applying strict filters to exclude false positives, we covered a total of 62,880 already annotated and de novo RNA editing sites among the samples, distributed in about 3000 genes (Figure 3A,C). A wide, uniform distribution of the identified RNA editing sites was observed among chromosomes, with chromosome 19 containing the greatest number of RNA editing sites, followed by chromosomes 16, 18 and 19 (Tables S2 and S3). The number and frequency of RNA editing varied between treated samples at considered time points. In total, 20,357 RNA editing sites, within 380 genes, were shared by control and all time points related treated RPE cells. Then, 31,324 RNA editing sites were found only in the control samples, 6637 were found only in RPE cell samples after 3 h of treatment, and 4562 were detected only in RPE cells after 6 h from A2E exposure (Figure 3B,D). 

### 3.4. Characterization and Distribution of Known RNA Editing Sites across Different Genomic Regions

**Figure 4:**
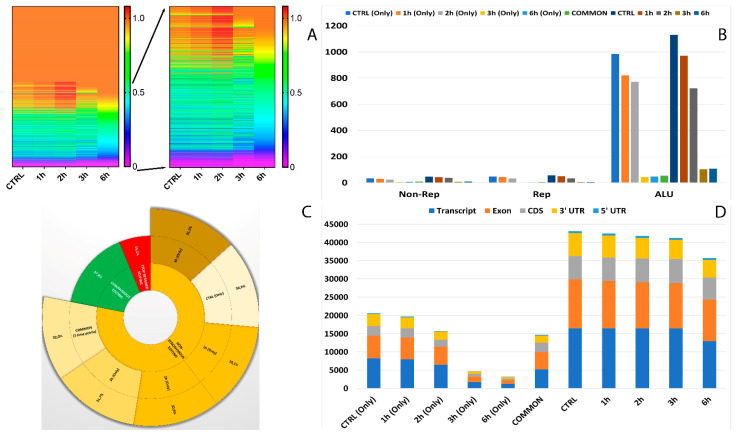
Editome known signatures during A2E exposure. (**A**) A heat map of the editing frequency from known sites that are particularly edited across A2E treatment. The color (from purple to red) indicates the editing frequency (from 0 to 1) for a given site (row) in a specific time point (column). The sub-heat map on the right underlines only the sites which changed during the treatment. (**B**) The proportion of known editing sites within ALU sequences, other repetitive elements, and outside SINEs across different time points. (**C**) The proportion of the non-synonymous shift in coding region affected by known editing sites. (**D**) The distribution of known editing sites across different genomic elements. Syn. = Synonymous. Non-Syn. = Non-Synonymous. Stop Ret. = Stop Retained. Comm. = Common. TPs = Time Points.

In total, 12 RNA editing types were detected, which included A-to-C, A-to-T, A-to-G, C-to-T, C-to-A, C-to-G, G-to-A, G-to-C, G-to-T, T-to-A, T-to-C, and T-to-G. Among all the editing types, 36.7% were of the canonical type (A-to-I and C-to-U) (Table S2). We next explored the alteration of RNA editing after A2E exposure and found different time-exposure related clustering of modified sites. As evidenced by hierarchical clustering, the biggest changes in editing frequency and number of events were reached after 3 h of treatment, becoming more gradual after 6 h exposure (Figure 4A).

**Figure 5:**
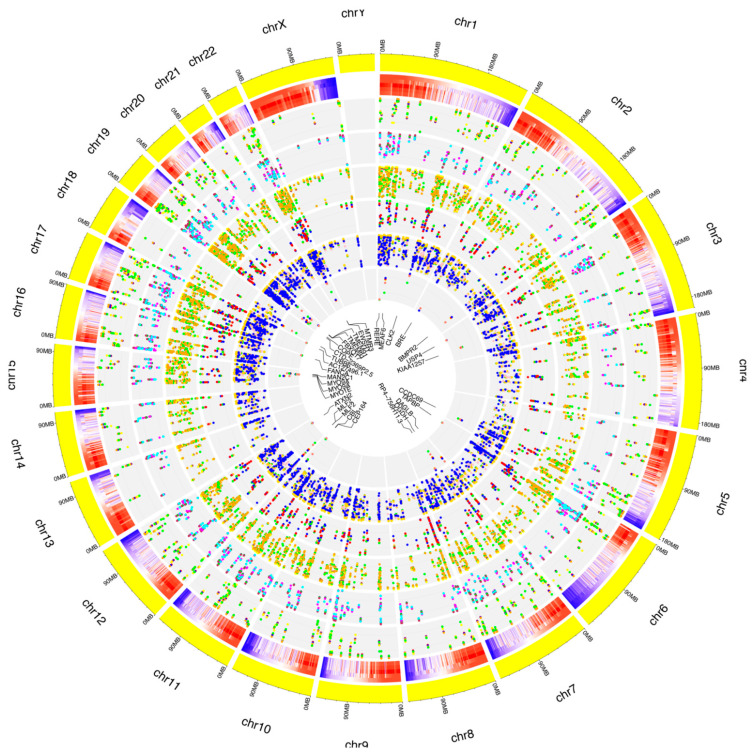
Distribution of known RNA editing sites from RPE cells across human chromosomes. The human genome is shown as a circle. For each chromosome, the position of the editing sites along with their average editing ratio (heatmap) is shown for different time points. From outside to inside: (1) N° of A-to-I only editing sites common to all considered time points and controls. The red, blue, orange, yellow and green dots indicate CTRL, 1 h, 2 h, 3 h and 6 h time points, respectively. (2) N° of C-to-U only editing sites common to all considered time points and controls. The purple, green, maroon, magenta and cyan dots indicate CTRL, 1 h, 2 h, 3 h and 6 h time points, respectively. (3) N° of editing sites within other-than-CDS and UTRs common to all considered time points and controls. The coral, navy, yellow, green and orange dots indicate CTRL, 1 h, 2 h, 3 h and 6 h time points, respectively. (4) N° of editing sites within CDS common to all considered time points and controls. The yellow, salmon, green, blue and red dots indicate CTRL, 1 h, 2 h, 3 h and 6 h time points, respectively. (5) N° of editing sites within UTRs common to all considered time points and controls. The green, orange, gold, pink and blue dots indicate CTRL, 1 h, 2 h, 3 h and 6 h time points, respectively. (6) N° of editing sites within repetitive elements common to all considered time points and controls. The yellow, red, blue, green and salmon dots indicate CTRL, 1 h, 2 h, 3 h and 6 h time points, respectively. For these editing sites, the related gene names are indicated.

Moreover, 32 of the RNA editing events changing frequency during the considered treatment occurred within short interspersed nuclear elements (SINEs), especially ALU elements. Interestingly, the number of editing modifications in ALU repeats drastically reduced during time exposure, especially if directly compared to controls (Figure 4B). The editing events within ALU repeats which showed the most differences in frequency were carried by *MEAF6, CCDC69, OGDH, MLF2, MYO1E, MYO9A, TUBB6, CTD-2369P.2.5, COPE, FBX017, TMEM91, EWSR1* and *MRMR3* (Figure 5).

One of the most interesting aspects related to RNA editing deals with its role in coding regions as a cause of non-synonymous shifts. The most non-synonymous events involved the same sites for all considered time points, with the highest percentage reached after the first 3 h of treatment (Figure 4C).

As shown in Figure 4D, the number of RNA editing sites varied among the different genomic regions. Most edited sites common to all considered time points were detected at transcript (35.8%) and exon (32%) levels, followed by CDS (17.4%) and 3′UTR (13.2%). The lowest number of differentially edited sites was detected within 5′UTR (1.5%). As already observed before, the number of editing events decreased during the advanced stages of treatment.

The known editing site characterization for events common to the whole treatment is summarized in the graphs in Figure 5. 

### 3.5. Characterization and Distribution of De Novo RNA Editing Sites across Different Genomic Regions

**Figure 6:**
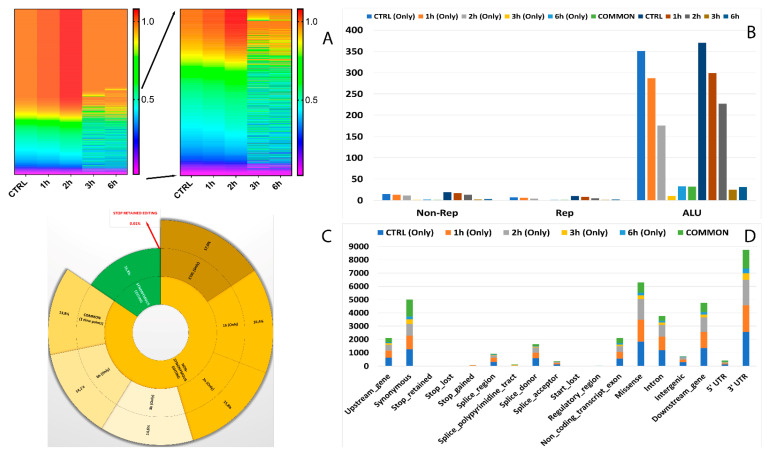
Editome de novo signatures during A2E exposure. (**A**) A heat map of the editing frequency from de novo sites that are particularly edited across A2E treatment. The color (from purple to red) indicates the editing frequency (from 0 to 1) for a given site (row) in a specific time point (column). The sub-heat map on the right underlines only the sites which changed during the treatment. (**B**) The proportion of de novo editing sites within ALU sequences, other repetitive elements, and outside SINEs across different time points. (**C**) The proportion of non-synonymous shift in coding region affected by de novo editing sites. (**D**) The distribution of de novo editing sites across different genomic elements. Syn. = Synonymous. Non-Syn. = Non-Synonymous. Stop Ret. = Stop Retained. Comm. = Common. TPs = Time Points.

The characterization of de novo RNA editing events foresaw the detection of 12 different types already described for known events, with 35.1% belonging to the canonical A-to-I and C-to-U (Table S3). This time, the hierarchical clustering highlighted two blocks: the first, with an increase in editing site frequency from 3h to 6h of treatment, and a wider second one with a huge decrease in editing site events during the same time points (Figure 6A). As already evidenced for known sites, the numbers of editing events decreased during the advanced stages of treatment.

**Figure 7:**
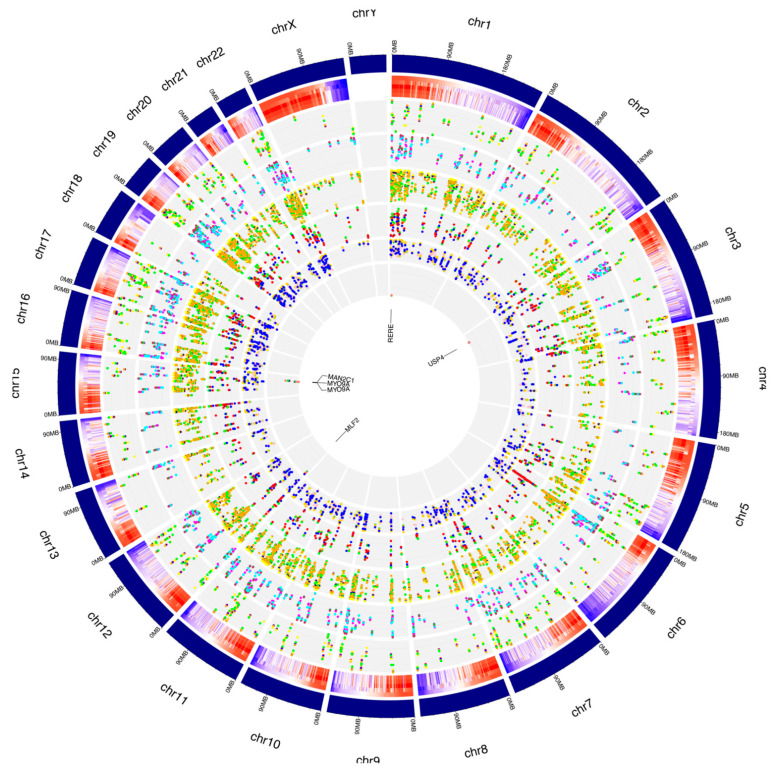
Distribution of de novo RNA editing sites from RPE cells across human chromosomes. The human genome is shown as a circle. For each chromosome, the position of the editing sites along with their average editing ratio (heatmap) is shown for different time points. From outside to inside: (1) N° of A-to-I only editing sites common to all considered time points and controls. The red, blue, orange, yellow and green dots indicate CTRL, 1 h, 2 h, 3 h and 6 h time points, respectively. (2) N° of C-to-U only editing sites common to all considered time points and controls. The purple, green, maroon, magenta and cyan dots indicate CTRL, 1 h, 2 h, 3 h and 6 h time points, respectively. (3) N° of editing sites within other-than-CDS and UTRs common to all considered time points and controls. The coral, navy, yellow, green and orange dots indicate CTRL, 1 h, 2 h, 3 h and 6 h time points, respectively. (4) N° of editing sites within CDS common to all considered time points and controls. The yellow, salmon, green, blue and red dots indicate CTRL, 1 h, 2 h, 3 h and 6 h time points, respectively. (5) N° of editing sites within UTRs common to all considered time points and controls. The green, orange, gold, pink and blue dots indicate CTRL, 1 h, 2 h, 3 h and 6 h time points, respectively. (6) N° of editing sites within repetitive elements common to all considered time points and controls. The yellow, red, blue, green and salmon dots indicate CTRL, 1 h, 2 h, 3 h and 6 h time points, respectively. For these editing sites, the related gene names are indicated.

Curiously, only six of the RNA editing events varying their frequency during A2E treatment occurred within SINEs, with the most different frequencies shown by *MAN2C1* (AluSx) and *MLF2* (AluSc) (Figure 7). Intriguingly, the number of editing modifications in ALU repeats showed an opposite trend if compared to the same data for known editing sites, with an increase from 3h to 6h (Figure 6B).

Interestingly, also for de novo analysis, most non-synonymous editing involved the same sites for all considered time points but showed a constant decrease from start to end of treatment, with the lowest percentage reached after 6 h of A2E exposure (Figure 6C). 

The VEP prediction permitted us to better detail the number of differences of RNA editing sites among the various genomic regions. Most edited sites common to all considered time points harbored 3′UTR (25.1%) and synonymous (22.6%) variants, followed by missense (13.8%) and downstream (12.3%) ones. The lowest number of differentially edited sites was detected at regulatory region and stop retained level, with only one editing site changed (Figure 6D).

The de novo editing site characterization for events common to the whole treatment is summarized in the graphs in Figure 7. 

### 3.6. Editing Site Comparison between Control and Treated RPE Cells Highlighted Pathways Involving Surface Protein as Most Edited

**Figure 8:**
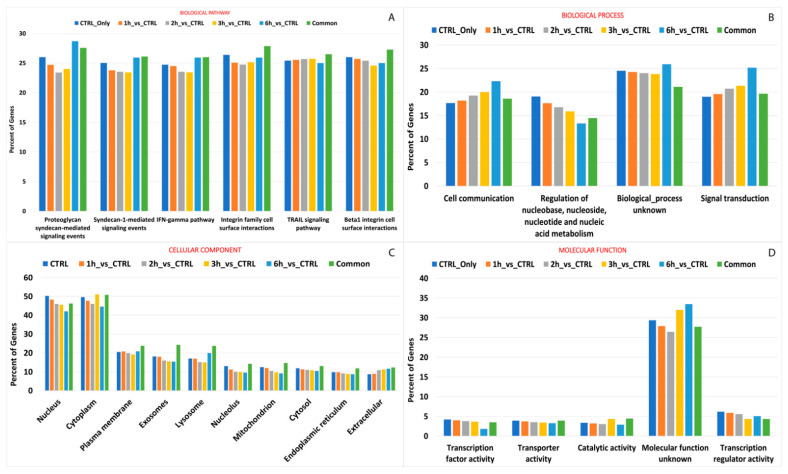
GO and KEGG enrichment of genes with differential editing levels. The vertical bar plots show the percentage of genes involved in top ranked pathway enrichment analyses, providing hierarchical relationships for the gene products based on biological pathway (**A**) and process (**B**), cellular component (**C**) and molecular function (**D**). Details on ranking criteria can be found in the text.

Most editing events that occurred during the whole treatment, especially after 3 h and 6 h from treatment, were carried by genes encoding protein products acting mainly within cytoplasm (n° edited genes_common_ = 1059, n° edited genes_3h_ = 194, n° edited genes_6h_ = 107), and involved in signal transduction (n° edited genes_common_ = 462, n° edited genes_3h_ = 94, n° edited genes_6h_ = 70). Interestingly, the editing events that occurred during A2E exposure seemed to alter the catalytic activity of many enzymes (n° edited genes_common_ = 104, n° edited genes_3h_ = 19, n° edited genes_6h_ = 93), as well as transcription regulation of specific genes after 3 h (n° edited genes_3h_ = 19) or 6 h from the treatment (n° edited genes_6h_ = 14). More differences among considered time points were evidenced regarding biological pathways. Common edited genes were mainly involved in integrin family cell surface interactions (n° edited genes_common_ = 285), while the tumor necrosis factor related apoptosis-inducing ligand (TRAIL) signaling pathway and proteoglycan syndecan-mediated signaling involved the highest number of editing events after 3 and 6 h from treatment start, respectively. Details on edited gene pathway enrichment are available in Figure 8 and Table S4.

### 3.7. Effects of RNA Editing Sites on miRNA–RNA Interactions

**Figure 9:**
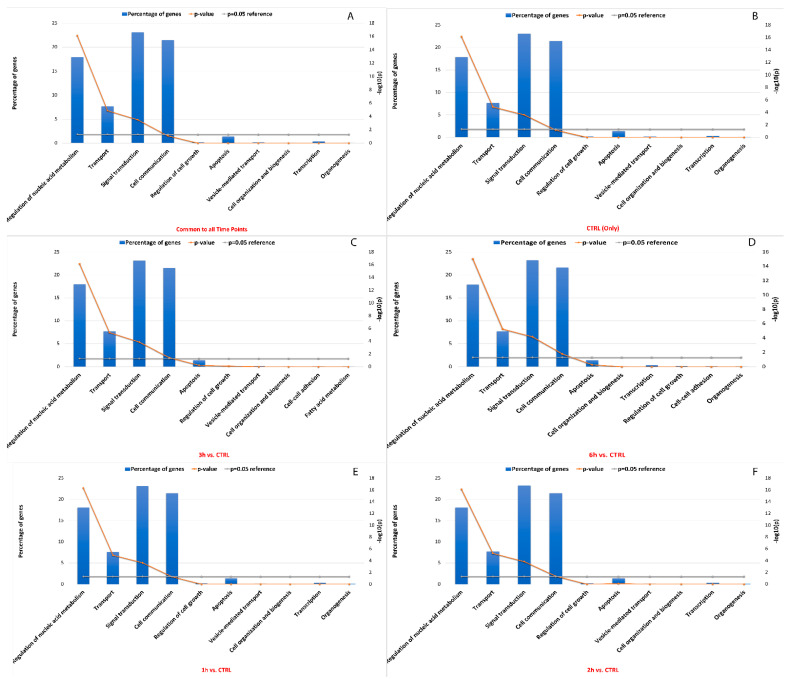
GO and KEGG enrichment of miRNA target genes with the highest number of editing sites. The vertical bar plots show the percentage of miRNA target genes involved in top ranked pathway enrichment analyses, considering genes common to all time points (**A**), only in the control samples (**B**), and in the other time points (**C**–**F**). The significance of the hypergeometric test (−log_10_ of *p*-value) is represented by the broken orange line. The first five pathways showed the highest number of miRNA target genes statistically associated.

Like miRNA binding site single nucleotide polymorphisms (SNPs), RNA editing sites within miRNA binding sites may impact interactions between miRNA and target RNA. Approximately 19% between known and de novo RNA editing sites were located within 3′ UTR, which included sites common to all time points that were predicted to change the binding capacity of 359 miRNAs towards 9654 target genes. In detail, a global decrease in edited miRNA binding sites was seen, as well as their related targets (n° miRNA edited binding sites_CTRL_ = 354, n° target genes_CTRL_ = 9653, n° miRNA edited binding sites_3h_ = 298, n° target genes_3h_ = 9444, n° miRNA edited binding sites_6h_ = 280, n° target genes_6h_ = 9330). According to enrichment analysis, the impacted target genes were mainly involved in signal transduction (22.5%), cell communication (21%), regulation of nucleic acid metabolism (18%), transport (7.5%) and apoptosis (2%) (Figure 9). Interestingly, it was shown that the Bonferroni corrected significance of the hypergeometric test generally increased from the statistical analysis involving 1h-related miRNA target genes to the 6h-related miRNA target genes involved in previously described pathways. More details are available in Table S5.

### 3.8. Deaminases Involved into Editing Showed a Global Up-Regulation of mRNAs

**Figure 10:**
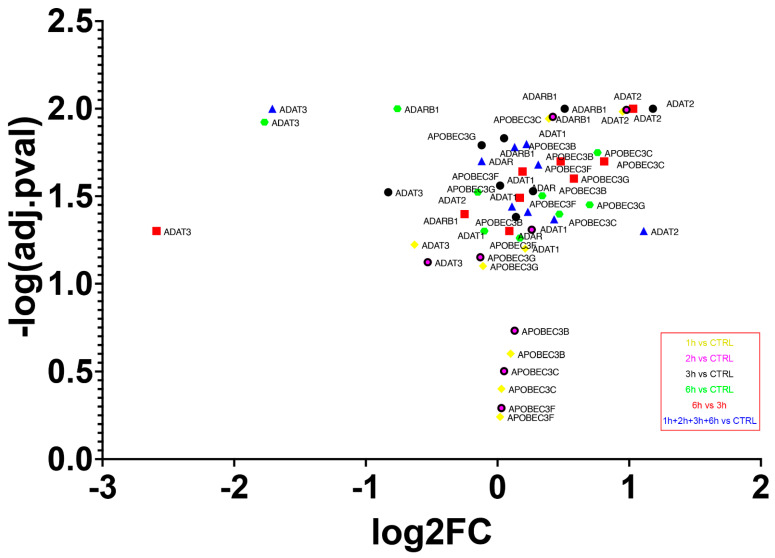
Expression of deaminase genes across A2E treatment. The volcano plot shows expression differences (FC) between different time points of deaminase encoding genes. All FCs for those genes were statistically significant (*p*-value ≤ 0.05).

The transcriptome experiment carried out permitted the analysis of expression levels of the main enzyme families involved in editing events. Overall, an increased expression of all editing enzymes was seen during each step of analysis, with the lowest growth from 3 h to 6 h of treatment (Figure 10). The highest expression level was reached by adenosine deaminase tRNA specific 2 (ADAT2) already from the first stages of A2E exposure (log_2_FC_3hvsCTRL_ = 1.18, *p*-value = 0.01), while the lowest was represented by another member of the ADAT family, adenosine deaminase tRNA specific 3 (ADAT3), but during the late stages of treatment (log_2_FC_6hvsCTRL_ = −2.59, *p*-value = 0.05). The canonical A-to-I editors, instead, showed a more gradual variation, with the highest expression changes reached by adenosine deaminase RNA specific (ADAR) during the last 6 h of exposure, and with an oscillatory trend manifested by ADAR2 (also known as ADARB1). Finally, the cytidine deaminases within the activation induced cytidine deaminase/apolipoprotein B editing complex (AID/APOBEC) family, mainly responsible for C-to-U mRNA editing, were all overexpressed during the whole treatment, with the highest peak reached by APOBEC3C after 6 h of A2E exposure (log_2_FC_6hvsCTRL_ = 0.81, *p*-value = 0.02). The deaminases selected mRNAs were validated by qRT-PCR analysis and the obtained expression profiles were similar to the results of transcriptome analysis (data not shown). Moreover, ANOVA statistics, conducted to compare means among multiple groups, highlighted high significance (*p*-values < 0.05).

## 4. Discussion

Eukaryotic organisms are characterized by complex transcriptomes whose regulation is critical for all cellular processes and homeostasis [62]. The dynamicity of transcriptomes is based on greatly modulated posttranscriptional mechanisms, such as alternative splicing and RNA modifications [63]. RNA editing is taking on a pivotal role in promoting transcriptome diversity and fine-tuning gene expression [64].

With the growing adoption of genome re-sequencing and RNA-Seq technologies, a considerable number of RNA editing sites in the genome of several animal species, Homo sapiens included, have been identified [64]. Currently, RNA editing in physiological and pathological human retinas remains quite unknown, and it could become clinically relevant in many diseases, such as age-related macular degeneration (AMD), diabetic retinopathy, and other retinal degenerations [65,66]. In the present study, we carried out a comprehensive profiling of RPE cells treated with A2E during a follow-up of four time points (1 h, 2 h, 3 h and 6 h) after exposure and compared them to untreated time zero controls. Using strand-specific RNA-Seq datasets with high sequencing depth and coverage, together with a complex multi-alignment data analysis pipeline, we were able to capture unprecedented editing events with low editing levels and in low depth regions, focusing on RNA changes during RPE cell death induced by the toxic effects of A2E. 

To our knowledge, this is the first systematic study of RNA editing in human retinal cells. We detected a total of 62,880 already annotated and de novo RNA editing sites throughout all time-related samples. Approximately 20,000 RNA editing sites were shared by control and both 3 h and 6 h treated RPE cells, and a global decrease in editing events was seen from start to end of A2E exposure. Then, 31,324 RNA editing sites were found only in the control samples, 6637 were found only in RPE cell samples after 3 h of treatment, and 4562 were detected only in RPE cells after 6h from A2E exposure, which suggested that the induced oxidative stress could heavily impair RNA editing activity. 

Twelve types of RNA editing were identified, among which A-to-I and C-to-U were identified as being the canonical RNA editing events, accounting for about 36% of all identified sites, whereas the remaining RNA editing types were the non-canonical ones [67]. A-to-I RNA editing events, one of the most common types of frequent editing events in mammals, are mediated by the ADAR family of enzymes [68]. In particular, ADAR1 and ADAR2 are known to be ubiquitously expressed and catalytically active, while ADAR3 is considered to be inactive [69]. No data were previously available on ADAR and ADAT family members in the retina, while only two works discussed APOBEC-mediated RNA editing within the eye. Leonardi et al. found that APOBEC3A was expressed at very high levels in some conjunctival samples, and ADAR1 was more specifically expressed in the cornea, as a consequence of SARS-CoV-2 infection [70]. The work realized by Xiao et al. is very interesting, where the authors found an early overexpression of APOBEC subunit 1 (APOBEC1) in mice retinas treated with N-methyl-d-aspartate (NMDA) plus epidermal growth factor (EGF). It was hypothesized that neurotoxicity induced APOBEC1 may promote cell cycle reentry of retinal Müller cells via DNA demethylation, manifesting a potential neural regeneration [71]. Our results showed that expression of all families’ editing enzymes were mainly up-regulated during the whole oxidative stress induction, with only ADAT3 down-regulated. Thus, with regard to already described editing site numbers, it could be hypothesized that editing activity and number of different editing events are not equally stage specific. Probably, the former increase constantly during the entire A2E exposure, but act on less genes with reduced frequencies, possibly due to a general compromission of several molecular pathways towards cell death. 

Our data revealed that a significant number of RNA editing events occurred in the coding regions, followed by intron and regulative ones, also in repetitive elements, suggesting that RNA editing may control nuclear retention and alternative splicing. In mammals, canonical editing frequently occurs within Alu repeat elements, which are mammal-specific SINEs [72]. It is already known that Alu RNA mediated cytotoxicity may induce RPE cell death by apoptosis through the accumulation of P3Alu transcripts, subsequent activation of the ERK1/2 signaling pathway, and the assembly of NLRP3 inflammasome [73]. Furthermore, it was established that oxidative stress causes Alu RNA accumulation via PIWIL4 sequestration in cytoplasmic stress granules [74]. Our results seem to confirm such results, highlighting that the highest number of ALU edited sites belonged to genes (*MEAF6, CCDC69, OGDH, MLF2, MYO1E, TUBB6, CTD-2369P.2.5, COPE, FBX017, TMEM91, EWSR1, MTMR3, MAN2C1*) involved in the “intracellular anatomical structure” molecular pathways (GO:0005622), especially in the cytoplasm and nucleus. 

Annotation of the edited genes with missense mutations permitted us to speculate on a possible neovascularization of retinal tissue, with induced apoptosis mediated by the extracellular matrix (ECM) and surface protein impaired signaling. Such pathways were already shown as possibly involved in RPE oxidative stressed cells, as already evidenced after the toxicity induced by A2E [31,75]. Common edited genes were mainly involved in integrin family cell surface interactions, already known to be involved in the cell-to-cell adhesion and signal transduction mediated by the ECM [76,77]. Integrins are transported into retinal ganglion cell axons in the retina and are probably needed for regeneration of compromised axons [78]. The TRAIL signaling pathway and proteoglycan syndecan-mediated signaling are involved in the highest number of editing events after 3 and 6 h from treatment start, respectively. TRAIL is well known to induce apoptosis in different cell types, and recently its role in increasing retinal neovascularization (NV) during oxygen-induced retinopathy has been evaluated [79]. Thus, it seems quite plausible that after 3 h from A2E exposure the TRAIL pathway might represent the most likely candidate to induce apoptosis in RPE cells. Shortly after, the syndecan signaling pathway became the one with the most edited genes, reaching the highest number of editing events after 6 h from treatment start. Thus, an alteration of syndecan related signaling might enforce the TRAIL-dependent NV, already recently evaluated [80,81,82,83], with a possible increase in the apoptotic cascade. 

In addition to missense editing sites, several RNA editing sites detected in our study were identified in UTRs of different transcripts, especially 3′UTR, and might alter miRNA-mediated posttranscriptional gene silencing and expression. The role of miRNAs in oxidative stress induced RPE cells is well described in literature [84], but no data are available on editing of their binding sites within target genes. According to the GO and KEGG analysis, such target genes were enriched in some critical pathways already considered as the main players in retinal cells degeneration, such as signal transduction and cell communication, with the final consequence of induced apoptosis. Based on these results, RNA editing sites within 3′UTRs may exert *cis*-regulatory impact on gene expression by influencing mRNA degradation and stabilization. In particular, the detection of RNA editing sites potentially related to miRNA activity on *LRAT* gene, fundamental for the esterification of all-*trans*-retinol to all-*trans*-retinyl esters and already associated in carcinomas [85] was interesting [86]. These editing events might alter both “Metabolism” and “Energy” pathways, albeit not fully statistically significant (Table S5). However, data on miRNA at 3h and 6h showed a different behavior from the rest of the editing and might deserve further investigation.

## 5. Conclusions

Recent advances in RNA-editing research have led to the creation of several RNA editing resources for humans. However, a database of RNA editing for the human retina is currently lacking. Thus, we created a comprehensive and dynamic atlas of the RNA editome in RPE cells across four different A2E exposure time points (1 h, 2 h, 3 h and 6 h) after the basal one. To our knowledge, this is the first genome-wide atlas of the RNA editome of the human retina. We identified different clusters of RNA editing sites associated with ECM and vascularization alterations, with consequent induction of RPE cell apoptosis. However, it has to be underlined that obtained results were produced with the condition of huge cell death at 6h from treatment. Thus, further experiments will be performed with a less dramatic stress stimulus and then progress towards an irreversible situation, to propose a combined functional assessment of the most relevant RNA editing which can eventually be made reversible upon intervention. At the same time, it will be useful to improve knowledge on the specific role of editing regulation on identified molecular pathways.

In conclusion, our present work provides information regarding the dynamic landscape of RNA editing during an experiment of induced oxidative stress in RPE cells, which might be useful for biomedical researchers who study retinal degeneration mechanisms to find new molecular markers.

## Figures and Tables

**Table 1:** Aligner parameters used in the analysis. The first point refers to genome indexing creation, while the second to the read mapping task. All other arguments are set to default if not specified. reference.fa = reference genome in FASTA format. sequence_R1.fq and sequence_R2.fq = input paired-end reads.

ALIGNER	COMMAND LINE USED
BWA-MEM	bwa index reference.fa bwa mem -t 4 reference.fa sequence.fastq > alignment.sam
CLC GENOMICS WORKBENCH	index automatically produced within software No Command Line, but GUI, with the following parameters: Match score = 1; Mismatch cost = 2; Cost of insertion and deletions = Linear gap cost; Insertion cost = 3; Deletion cost = 3; Length fraction = 0.5; Similarity fraction = 0.8; Global alignment = no; Auto-detect paired distances = yes; Non-specific match handling = Map randomly
HISAT2	hisat2-build -f reference.fa reference hisat2 -f -x reference-1 sequence_R1.fq −2 sequence_R2.fq -S alignment.sam
STAR	STAR --runThreadN 4 --runMode genomeGenerate --genomeDir Genome_data/star\ --genomeFastaFiles Genome_data/reference.tar.gz STAR --readFilesIn sequence.fastq\ alignIntronMax 1\ --genomeLoad LoadAndKeep\ --genomeDir /path/to/genomeFasta/\ --runThreadN 4\ --outStd SAM > alignment.sam
RASER	./raserIdx -r reference.fa mRNA_reference.fa -l reference.gtf -x reference.ridx ./raser -x reference.ridx -i1 sequence_R1.fq -i2 sequence_R2.fq -o alignment.sam -b 0.03
TOPHAT2	bowtie2-build -f reference.fa reference tophat -o alignment reference sequence.R1.fq sequence.R2.fq

## Data Availability

All data supporting results are available as Supplementary Materials.

## Supplementary Materials

The following supporting information can be downloaded at: https://www.mdpi.com/article/10.3390/antiox11101967/s1, Table S1: List of primers used for qRT-PCR data validation, Table S2: Distribution and features of identified known editing sites across time points, Table S3: Distribution and features of identified de novo editing sites across time points, Table S4: Pathway enrichment of identified editing sites across time points, Table S5: miRNA edited target genes enrichment.
